# Women's experiences of wearing therapeutic footwear in three European countries

**DOI:** 10.1186/1757-1146-3-23

**Published:** 2010-10-08

**Authors:** Anita E Williams, Christopher J Nester, Michael I Ravey, Anke Kottink, Morey-Gaspar Klapsing

**Affiliations:** 1 Directorate of Prosthetics, Orthotics and Podiatry, University of Salford, Frederick Road, Salford, UK; 2 Centre for Health, Sport and Rehabilitation Sciences, University of Salford, Salford, UK; 3 School of Nursing, University of Salford, Frederick Road, Salford, UK; 4 Roessingh Research and Development, Enschede, The Netherlands; 5 INESCOP, Elche, Spain

## Abstract

**Background:**

Therapeutic footwear is recommended for those people with severe foot problems associated with rheumatoid arthritis (RA). However, it is known that many do not wear them. Although previous European studies have recommended service and footwear design improvements, it is not known if services have improved or if this footwear meets the personal needs of people with RA. As an earlier study found that this footwear has more impact on women than males, this study explores women's experiences of the process of being provided with it and wearing it. No previous work has compared women's experiences of this footwear in different countries, therefore this study aimed to explore the potential differences between the UK, the Netherlands and Spain.

**Method:**

Women with RA and experience of wearing therapeutic footwear were purposively recruited. Ten women with RA were interviewed in each of the three countries. An interpretive phenomenological approach (IPA) was adopted during data collection and analysis. Conversational style interviews were used to collect the data.

**Results:**

Six themes were identified: feet being visibly different because of RA; the referring practitioners' approach to the patient; the dispensing practitioners' approach to the patient; the footwear being visible as different to others; footwear influencing social participation; and the women's wishes for improved footwear services. Despite their nationality, these women revealed that therapeutic footwear invokes emotions of sadness, shame and anger and that it is often the final and symbolic marker of the effects of RA on self perception and their changed lives. This results in severe restriction of important activities, particularly those involving social participation. However, where a patient focussed approach was used, particularly by the practitioners in Spain and the Netherlands, the acceptance of this footwear was much more evident and there was less wastage as a result of the footwear being prescribed and then not worn. In the UK, the women were more likely to passively accept the footwear with the only choice being to reject it once it had been provided. All the women were vocal about what would improve their experiences and this centred on the consultation with both the referring practitioner and the practitioner that provides the footwear.

**Conclusion:**

This unique study, carried out in three countries has revealed emotive and personal accounts of what it is like to have an item of clothing replaced with an 'intervention'. The participant's experience of their consultations with practitioners has revealed the tension between the practitioners' requirements and the women's 'social' needs. Practitioners need greater understanding of the social and emotional consequences of using therapeutic footwear as an intervention.

## Background

Therapeutic footwear is provided for people with rheumatoid arthritis (RA) who cannot wear mainstream retail footwear due to foot pathology [1,2] and foot pain [3]. Recent guidelines [4,5] and research [6,7] support the use of therapeutic footwear in reducing foot pain and improving mobility. However, patients have long reported dissatisfaction with this footwear [8-11] resulting in them being described as; "...shoes in the cupboard" [12]. Authors of more recent research and reports [13-18] both in the UK and the Netherlands have recommended changes in service provision and footwear design. However, it is still perceived that the potential benefits of wearing therapeutic footwear are still negated by the patients' choice not to wear them.

People with RA can have complex foot problems and therefore the factors that influence footwear choices are potentially complex. Previous research has explored personal accounts of using therapeutic footwear [16], evaluation of its 'usability' [17] and patient expectations of it [18]. However, questions still remain in relation to our understanding of the persons' feelings about the process of receiving this footwear from the point of referral, through to its provision and how these experiences relate to the patients subsequent choice to use it or not.

Previous work by the authors [16] has indicated that women have more complex needs in relation to the appearance of footwear in comparison to men. For the men, it was identified that the replacement of existing footwear is not as noticeable as it closely resembles the footwear that they would normally wear. Contrastingly, for the women, the loss of femininity imposed by the appearance of the footwear impacted on many facets of their lives.

Therefore, the aims of this study were to further explore women's experiences of the process of being referred for and being provided with therapeutic footwear and to gain further insight into factors contributing to their choice to wear therapeutic footwear or not. This is the first study to be carried carried out in three European countries (UK, the Netherlands and Spain). This was planned to explore potential differences in the patient's cultural attitudes to therapeutic footwear and in current service delivery.

## Method

This study adopted an interpretive phenomenological approach [19,20]. This is a research philosophy that considers the lived experiences of the person being researched and also the researcher's experiences through a process of reflexivity. The result is a 'fusion of horizons' [20] that produces a 'rich' and unique account of both the experience and the associated feelings and emotions. Conversational style interviews were used in order to explore the participants' personal experiences of receiving therapeutic footwear and their feelings about that experience.

Following ethical committee approval in the three countries, participants were purposively recruited from rheumatology and orthotic services by the researchers. This was to avoid selection bias from the service providers. The inclusion criteria were:

• Women diagnosed with rheumatoid arthritis [21].

• Provided with footwear for a minimum of six months before the study started, in order to have sufficient experience of both service delivery and therapeutic footwear use.

Women meeting these criteria were sent letters and information leaflets about the study. The first ten women responding from each country were recruited. The interviews were carried out in the UK (AW), the Netherlands (AK) and in Spain (AW with GMK translating). Both researchers had experience of therapeutic footwear from both a clinical and research context and further to this achieved agreement in their interview approach before the interviews took place.

Written consent was obtained and all participants agreed to proceed to the interview, which was recorded using a digital voice recorder. Participants were assured they could stop the interview at any time and withdraw from the study if they so wished. The participant's age, duration of RA and current therapeutic footwear use was documented. The researcher asked an initial question, "*Tell me about your experiences of specialist (therapeutic) footwear referral"*. Further probes were used if the conversation came to a halt, such as, "...how did you feel when your family saw the shoes?" and "... tell me what it was like when you collected your shoes from the hospital?"

Pseudonyms were used in interview transcripts to ensure confidentiality. Translation and transcription of the interviews was carried out by AK (the Netherlands) and GMK (Spain). Field notes from AW (UK and Spain) and AK (the Netherlands) supplemented the data from the translations.

The data analysis was carried out by one researcher (AW). Self-reflection on her previous clinical and research experience in the area of therapeutic footwear took place and continued throughout the interpretative process. This acknowledges that the interpretation of the participant's dialogue is enriched by the researcher's previous knowledge and experience. The result is that the new meanings that are revealed from the data are fused with previous understanding so that new knowledge and understanding is produced [22].

Thematic analysis was employed [23] with each transcript being read several times before significant statements and meanings were identified. These were then organised into main themes which formed the basis of the results. The themes were scrutinised by two academics (MR and CN) in relation to the transcripts to add to the reliability of the initial analysis. Exemplars from the transcripts were identified to support each of the themes. Presenting these to the reader reveals the 'authentic' nature of the experiences in relation to the identified themes and therefore supports the trustworthiness of the data [22].

## Results

Thirty women participated [10 UK, 10 the Netherlands (NL) and 10 Spain (SP)]. Participant demographics are detailed in Table 1.

**Table 1:** Participant demographics

	UK	Spain	The Netherlands
Age (yrs)	Mean 57 (SD 10.68)	Mean 57 (SD 6.01)	Mean 58 (SD 11.93)

Disease duration	Mean 15 (SD 6.72)	Mean 13 (SD 4.96)	Mean 15 (SD 5.16)

Foot pain (VAS)	Mean 8 (SD 1.14)	Mean 6 (SD 1.20)	Mean 7 (SD 1.25)

Significant statements and meanings from the dialogue were organised into six overarching themes.

### Theme 1 - Feet being visibly different because of Rheumatoid Arthritis

All the women stated their feet were visibly different to other women whom they considered 'normal'. This visible difference was how the feet looked and how they affected their ability to walk and function 'normally' because of foot pain. The women expressed concern about how others see them, their perceived loss of femininity, and the advancement of old age as "Rose" reveals;

".....well it shows in your face...the pain you know...maybe because you are stood on them...makes you self conscious as well...I look and feel like an old lady". Rose (UK)

The impact of their feet on close relationships was of concern to many, as highlighted by both Catalina and Lily;

"I feel awful and most of the time I feel bad about my appearance ...I don't feel feminine any more...my feet...well I don't show them...hide them as much as possible...I get sad when I see women with straight toes and well manicured nails and I think my husband will not like me any more...". Catalina (Spain)

"I can't walk normally... If you shuffle around people notice and (thinks for a time)........it shows in your face...pain shows in your face...makes you look old and I feel ...when I look in the mirror god what must my husband think..." Lily (UK)

Many statements about their feet revealed emotions such as frustration, anger, anxiety, loss and sadness. These were expressed both verbally, "I am *angry *that this has happened to me...", and in the way these statements were said, for example with a raised and angry voice during the interview.

### Theme 2 - The referring practitioners' approach to the patient

When compared with the women from the UK, the women from The Netherlands and Spain provided evidence of discussion between themselves and the referring practitioner (rheumatologist) before a referral for the therapeutic footwear was made;

"....I had an idea what the shoes would be like...he (the rheumatologist) told me ...even had pictures of them and then pictures of surgery... ...he was great and I didn't feel a problem when I said no to the shoes ...I think he understood the issues with the appearance of them...I am a smart lady...like dresses and he complemented me on that..." Reina (SP)

"My Rheumatologist was... well... understanding my problem very well. In the hospital they took some pictures of her feet and these were examined thoroughly to decide what would be the solution for the foot pain." Odette (NL)

In contrast, the women from the UK all reported that they passively agreed to the referral by their rheumatologists, as evidenced by Daisy's experience;

*"... I would have liked more choice as to whether to have the footwear in the first place... I felt I didn't have time to consider whether I wanted it or not....just....well...went along with what the doctor said*". Daisy (UK)

As in theme 1, the emotions of frustration, anger, anxiety, and sadness were in evidence in relation to the consultation, with 'lack of voice', lack of information, and lack of discussion and choice being the key issues for the women from the UK.

### Theme 3 -The dispensing practitioners' approach to the patient

It was generally expressed that the women had trust in the dispensing practitioners' skills. However, there was a mix of experiences in relation to the practitioners approach to the consultation, with some revealing both 'good' and 'bad' experiences. Some described the practitioner as being a 'nice person', which was linked to them visibly trying to provide acceptable footwear. However, many identified negative attributes, for example, being dismissive of the patients concerns and having poor communication skills and lack of demonstrable understanding invoked feelings of shame and anger, as those expressed by Daphne and Alison;

*"There was no discussion....if fact I don't really think it mattered what I thought ...just said I had difficult feet and that made me feel **ashamed **..." Daphne *(UK)

"I think generally there is not much understanding about how rheumatoid arthritis affects the person...well...we are people aren't we ....we are just not a pair of feet we have feeling... and sometimes I feel that they (practitioners) don't understand...that makes me **angry**..." Alison (UK)

However, a minority reported good experiences but this was more evident in the Netherlands;

"The person who helped was very kind and understood the foot problems. He also explained what he was doing all the time ... Every choice he had to make was reasoned out....this felt very good... " Femke (NL)

Interestingly, half the women expressed that they considered the timing of referral for this footwear coincided with coming to terms with the impact of RA on their lives, as Sierra identifies;

*"They helped me in a professional and cool way, which was OK. However, I had a strange feeling and **sadness **when I left, because I was in the process of acceptance of the problems that this arthritis puts into your life." *Sierra (SP)

In addition to a lack of 'voice' during the consultation with the dispensing practitioner these women expressed the emotions of shame, sadness and anger as identified in the exemplars from Alison, Femke and Sierra. The women's perception of the dispensing practitioners' lack of understanding and poor attitude appeared to reinforce both the negative view of their foot problems and the emotions that they expressed.

### Theme 4 - The footwear being visible to others

For the majority, the feelings about their feet were reinforced by the reaction of 'others' to their therapeutic footwear. Because of their footwear, they considered themselves as being visibly different to women of their own age, and this impacted on their self esteem. Again, the emotions of shame, sadness, and anger were expressed in relation to this footwear drawing attention to their feet;

"I wear very long trousers to cover up the shoes...I am **ashamed **of them but can't walk well without.....they are bulky and I don't want my friends to see them" Savanna (SP)

Further to this, many of the women viewed being given this footwear as the final and most symbolic marker of having a disease that impacts significantly on appearance and choice, as identified by both Karin and Yvonne;

"When you have RA, you get used to handing in a lot of things bit by bit. I didn't enjoy the **moment **of being given shoes. ..I was so disappointed... because every time you have to turn in a little bit when you have RA..." Karin (NL)

"...while wearing this footwear your illness becomes the area for attention... I don't deny my illness, but I don't want my illness to become me ...it takes a lot of things away from you. To be provided with orthopaedic shoes was the last step though." Yvonne (UK)

These statements are powerful, not only in revealing the negative feelings and emotions associated with RA, but also the notion that therapeutic footwear is the symbolic marker of RA. The significance and role that this footwear has in this respect may not be understood by practitioners.

### Theme 5 - Footwear influencing social participation

The loss of femininity and how the footwear impacts on the women's sexuality was further expressed through how it affects their participation in social activities. The few participants who had chosen to wear the footwear acknowledged that it improved their mobility, but that it also restricted social participation, as both Lena and Nadia revealed;

"...don't go out much but sometime makes me feel like crying and I panic when I do get an invite... I think oh gosh these boots....I was invited to a wedding and just sat at home and cried". Lena (UK)

"I felt very tearful the first time I had the shoes...and in shame...I didn't go out... I tried them with my clothes and I looked untidy..." Nadia (NL)

Activities that have the potential to increase independence were often restricted because the footwear was not suitable for seasonal changes and for some activities;

"... I can't and won't wear these in the summer... it's just too hot... I don't do out at all if my feet are bad rather than wearing these..." Margarita (SP)

"... I can't wear these to the gym I would look ridiculous - I have stopped going and that's not good for my joints... " Juliana (SP)

Again, the emotions of shame, sadness, and anger were identified as being associated with the impact of this footwear on the participants' activities. One of the women from the UK succinctly vocalises the impact of the footwear and the considered positives and negatives of wearing it;

"...these shoes are great...they improve my mobility but restrict my activities..." Daphne (UK)

### Theme 6 - The women's wishes for improving their experience

The women were vocal about what they needed to improve their experience. Information from their consultant rheumatologist, together with their needs being acknowledged, would have improved their understanding and aided their decision making as to whether to be referred or not. Nadia reveals the sadness she felt when she was not listened to;

"It is good to talk to someone who listens even though there is nothing that can be done...if you understand it helps ....I feel very sad that I wasn't listened to...if I had it might have been better." Nadia (NL)

Additionally, having their needs acknowledged was important throughout the process of being provided with the footwear. Yvonne reveals her experience of the 'fitter';

"I think that the fitter.... needs to listen to us more...I don't feel I was listened to about the footwear and now I feel guilty that I don't wear them...what a waste...that makes me angry." Yvonne (UK)

Acknowledgment that the women had unique knowledge of their own disease would have made them feel important. This in turn may have enhanced their experience and perhaps negate some of the 'bad' emotions felt by the women. Carol expresses an opinion about the importance of their experience of the disease being acknowledged;

"...you assume they know their job but we know our bodies don't we?...I know what will work....and it's not just a matter about what will work for our bodies ...it has to feel right.....look right and ...well its more about how we feel in the head isn't it?.." Carol (UK)

Further to this, the lack of a follow up appointment to review the footwear seemed to assign lack of value on both the footwear and the women themselves;

"There should be a review appointment some weeks after to check that I am doing OK ...I think they don't really care..." Juliana (SP)

This lack of value is further reinforced by their perception that practitioners have a poor level of understanding of RA and also undervalue its impact on the individual. Odette provides a solution to this by suggesting that;

"...more courses have to be organized to inform the clinical people what RA is doing with your body, what it takes away from you..." Odette (NL)

These women have clearly identified factors around the consultation with both the referring and dispensing practitioners. It was considered that these would have enhanced their experience of being assessed for and being provided with this footwear.

## Discussion

Through utilising an interpretive phenomenological approach, the women in this study have been able to voice their opinions on extremely important issues in relation to their footwear experiences, needs and self identity. This 'voice' is in contrast to their experience of the clinical situation. Additionally, this study adds to the body of knowledge about what is known from authors who have used quantitative methods of investigating the issues in relation to therapeutic footwear [9-12]. In contrast to these previous studies, this qualitative approach has allowed these women to reveal personal and emotive accounts of the meaning and impact of being provided with therapeutic footwear. Further to this they have demonstrated that they have clear opinions on what would potentially improve their experiences.

Despite the different countries and cultures, all the women revealed many aspects of their 'footwear experiences' with which they were not *satisfied*. However, they all expressed being *grateful*, even if this resulted in compromise with their footwear use, normal activities and their choice of clothes. This 'gratefulness' and compromise may be what 'patient satisfaction' questionnaires have measured and has resulted in a biased view of the true impact of therapeutic footwear in terms of women's priorities.

That the women focussed on their feet as being a visible marker of RA, highlights the inextricable liaison between feet, footwear and the creation of their sense of identity. Therapeutic footwear reinforces their emotions about their changing body, and how they are different to women of the same age, reinforcing their changed identities. This negative self image has been recognised previously in this patient group [24] but this appears to be infrequently acknowledged by the practitioners providing the footwear. Being forced to change their clothing styles in an attempt to disguise the footwear removes another choice in the lives of these women. Further to this it is seen as a final and symbolic marker of how RA impacts on their lives invoking strong and negative emotions.

The consultation with the referring practitioner is an opportunity to allow the patient to have informed choices. This opportunity is often missed for the women from the UK, who, in comparison with those from Spain and the Netherlands, behaved as passive recipients with no opportunity to reject the option of being referred for this footwear. This passive role appears to be perpetuated throughout the whole process of being referred and provided with this footwear. Therefore, the source of the problem could be perceived to be at the point of referral. It is evident that this reductionist experience increases the likelihood of women choosing not to wear the footwear once it has been supplied. Additionally the timing of referral may be important with some of the women reporting that the therapeutic footwear provision coincided with having to accept the impact of RA. Both the referring and dispensing practitioners need to be aware of where the patient is on their journey to acceptance of RA. If acceptance of RA has not occurred then they may not accept the footwear.

The footwear itself and its impact on restricting their choice in clothes could be seen as a contributing factor to reduced participation and social isolation which is evident in people with chronic disease [25,26]. In a previous study that included male participants' [16] they found the visual appearance of their footwear acceptable for their gender image and clothing choices. So this clearly is more of a problem for women and an approach by the dispensing practitioners that recognises and acknowledges this would be beneficial.

The focus of much of the women's dialogue was around the consultation with the dispensing practitioner. There was a mix of experiences in each of the countries and some individuals reporting both good and bad experiences. The body language of the practitioner, their poor attitude and lack of communication appeared to reinforce the women's negative feelings about the whole experience. The lack of opportunity or time for dialogue possibly reinforces that the balance of power lies with the dispensing practitioner. Further to this, some women expressed that they perceived the dispensing practitioner as having little or no knowledge of RA. Whether they did or not is not known but indicates the lack of acknowledgement from the practitioners about the impact of RA and the women's 'needs'. This lack of communication evident between the practitioner and these women detracts from the development of a therapeutic relationship whereby discussion and negotiation can support greater understanding for both. Patients are the experts in how the condition affects them and when this was acknowledged (mostly for the women from the Netherlands and Spain) then there was a sense of control in the decision making process from referral right through to the dispensing of the footwear. However, for the women from the UK it was clear that control was only regained by them through their choice of wearing or not wearing the footwear once it has been dispensed. Whilst there was some evidence and acknowledgement that the dispensing practitioners were experts in this footwear this was negated by failure to acknowledge that these women are experts in their own condition.

The relationship between these women and the dispensing practitioners was identified as vital in influencing whether they engaged in the use of therapeutic footwear. This relationship is described as a partnership in which sharing of information, support, and empathy is necessary to influence the patient's perceptions of control in a positive way [27-30].

Effective consultation skills should be integrated into training for all practitioners involved in the provision of therapeutic footwear. There is evidence to support the effectiveness of these consultation skills being taught in other areas [31,32]. This innovation in teaching and learning could be considered a good investment if it reduces the number of shoes not worn. Evaluation of the impact of this training on patient focussed outcomes could be the subject of further research.

There were challenges in this study in relation to the necessity of using two researchers and additionally a translator in Spain. To ensure a consistent approach, the researchers achieved consensus before the interviews took place. There was also the potential to miss the emotions and feelings assigned to the dialogue of the participant's accounts. However, this was overcome by the researcher (AW) making field notes on the emotions expressed during the interviews.

This study could be critiqued for the low number of participants and hence it could be stated that it has poor generalisability. However, this is not the aim of this methodological approach and importantly, this work has revealed new information that has the potential to improve clinical practice and therefore the experiences of women with RA wearing therapeutic footwear. All the women expressed a desire to help improve the footwear, the services that provide it, both for themselves and other women in the same situation. As Yvonne (UK) expressed,

*"Thank you for listening ...it is the first time anyone has done this... I really hope that this research helps improve the service for others in my position"*.

Therefore it is our duty, as members of the clinical and research community that we do listen. When a more patient centred approach is adopted in all aspects of the referral and dispensing process we should be able to evaluate whether this footwear not only 'improves their mobility...' but also maintains participation in all life's activities.

## Conclusion

Previous studies have not explored the depth of women's feelings and opinions of their therapeutic footwear experiences or attempted to investigate this in different countries. This unique study, carried out in three countries has revealed emotive and personal accounts of what it is like to have an item of clothing replaced with an 'intervention' that is viewed by these women as a final and symbolic marker of the disease. The participant's experience of their consultations with practitioners has revealed the tension between the practitioners' requirements and the women's 'social' needs. When a patient focussed approach was evident, these women had a much better experience of being provided with the footwear. Practitioners need greater training in patient-focused consultation styles in order to understand the social and emotional consequences of using therapeutic footwear as an intervention.

## Competing interests

The authors declare that they have no competing interests.

## Authors' contributions

AW conceived the study design, interpreted the findings and drafted the manuscript. AK and AW (with translation by GMK) interviewed the participants in The Netherlands and Spain respectively. All authors read and approved the final manuscript.
